# High Nuclease Activity of Long Persisting *Staphylococcus aureus* Isolates Within the Airways of Cystic Fibrosis Patients Protects Against NET-Mediated Killing

**DOI:** 10.3389/fimmu.2019.02552

**Published:** 2019-11-05

**Authors:** Susann Herzog, Felix Dach, Nicole de Buhr, Silke Niemann, Jannik Schlagowski, Diego Chaves-Moreno, Claudia Neumann, Jonas Goretzko, Vera Schwierzeck, Alexander Mellmann, Angelika Dübbers, Peter Küster, Holger Schültingkemper, Ursula Rescher, Dietmar H. Pieper, Maren von Köckritz-Blickwede, Barbara C. Kahl

**Affiliations:** ^1^Institute of Medical Microbiology, University Hospital Münster, Münster, Germany; ^2^Interdisciplinary Center for Clinical Research, Münster, Germany; ^3^Institute of Physiological Chemistry, University of Veterinary Medicine Hannover, Hanover, Germany; ^4^Helmholtz Center for Infection Research, Helmholtz Association of German Research Centers (HZ), Brunswick, Germany; ^5^Center for Molecular Biology of Inflammation (ZMBE), Institute of Medical Biochemistry, University of Münster, Münster, Germany; ^6^Institute of Hygiene, University Hospital Münster, Münster, Germany; ^7^Department of Pediatrics, University Hospital Münster, Münster, Germany; ^8^Department of Pediatrics, Clemenshospital, Münster, Germany

**Keywords:** Cystic fibrosis, *Staphylococcus aureus*, neutrophil extracellular traps, nuclease, adaptation, NET-mediated killing

## Abstract

*Staphylococcus aureus* is one of the first and most prevalent pathogens cultured from the airways of cystic fibrosis (CF) patients, which can persist there for extended periods. Airway infections in CF patients are characterized by a strong inflammatory response of highly recruited neutrophils. One killing mechanism of neutrophils is the formation of neutrophil extracellular traps (NETs), which capture and eradicate bacteria by extracellular fibers of neutrophil chromatin decorated with antimicrobial granule proteins. *S. aureus* secretes nuclease, which can degrade NETs. We hypothesized, that *S. aureus* adapts to the airways of CF patients during persistent infection by escaping from NET-mediated killing via an increase of nuclease activity. Sputum samples of CF patients with chronic *S. aureus* infection were visualized by confocal microscopy after immuno-fluorescence staining for NET-specific markers, *S. aureus* bacteria and overall DNA structures. Nuclease activity was analyzed in sequential isogenic long persisting *S. aureus* isolates, as confirmed by whole genome sequencing, from an individual CF patient using a FRET-based nuclease activity assay. Additionally, some of these isolates were selected and analyzed by qRT-PCR to determine the expression of *nuc1* and regulators of interest. NET-killing assays were performed with clinical *S. aureus* isolates to evaluate killing and bacterial survival depending on nuclease activity. To confirm the role of nuclease during NET-mediated killing, a clinical isolate with low nuclease activity was transformed with a nuclease expression vector (pCM28*nuc*). Furthermore, two sputa from an individual CF patient were subjected to RNA-sequence analysis to evaluate the activity of nuclease *in vivo*. In sputa, *S. aureus* was associated to extracellular DNA structures. Nuclease activity in clinical *S. aureus* isolates increased in a time-and phenotype-dependent manner. In the clinical isolates, the expression of *nuc*1 was inversely correlated to the activity of *agr* and was independent of *saeS*. NET-mediated killing was significantly higher in *S. aureus* isolates with low compared to isolates with high nuclease activity. Importantly, transformation of the clinical isolate with low nuclease activity with pCM28*nuc* conferred protection against NET-mediated killing confirming the beneficial role of nuclease for protection against NETs. Also, nuclease expression in *in vivo* sputa was high, which underlines the important role of nuclease within the highly inflamed CF airways. In conclusion, our data show that *S. aureus* adapts to the neutrophil-rich environment of CF airways with increasing nuclease expression most likely to avoid NET-killing during long-term persistence.

## Introduction

Cystic fibrosis (CF) is an autosomal recessive disease with mutations in the CF transmembrane conductance regulator (CFTR) gene causing a life-limiting multisystemic disease (1). Due to CFTR mutations, a dehydrated thickened airway surface fluid impairs mucociliary clearance and leads to chronic recurrent bacterial airway infections, which result in the decline of lung function and a reduced life expectancy (1, 2). *Staphylococcus aureus* is one of the most common bacterial pathogens in young CF patients that can persist for several years thereby causing high inflammatory responses in CF patient airways (3–5).

One of the hallmarks of CF lung disease is an exaggerated airway inflammation caused by excessive recruitment of dysfunctional neutrophils and accumulation of pro-inflammatory agents, which in turn fail to eradicate bacteria (6). Within the airways, neutrophils try to kill pathogens by different killing mechanisms such as phagocytosis with the release of oxidants and degrading enzymes during degranulation, and the formation of neutrophil extracellular traps (NETs) (7), which were previously described to be abnormal in CF (8, 9). In detail, bacterial digestion in the neutrophilic phagolysosome in CF is reduced by the lack of membranous chloride transport due to CFTR mutations causing defective intraphagolysosomal HOCL production and reduced chlorination of bacterial proteins (9). Moreover, cytosolic pH acidifies and leads to a massive release of antimicrobial enzymes from granules such as myeloperoxidase and neutrophil elastase and lactoferrin (10). The high concentration of neutrophilic defense peptides contributes additionally to the destruction of airway and lung tissue in CF (11, 12). It has been shown, that in the context of CF lung disease, NET formation by neutrophils is enhanced (13). Besides antimicrobial components of the neutrophil granules, NETs consist of extracellular DNA fibers released by chromatin decondensation and subsequent rupture of the nuclear membrane to capture and kill various pathogens (7, 11). Recently, the presence of NETs within CF airways has been shown and has been associated with poor pulmonary function assumingly driven by NET-mediated inflammation and increased amounts of thickened mucus (14, 15). *S. aureus* is not only a potent inducer of NETs (7, 16), but has also the potential to degrade NETs by the secretion of nuclease (17). We hypothesized, that in the airways of CF patients *S. aureus* will adapt to NET-mediated killing by increasing nuclease activity in long-persisting isolates. First, we used fresh sputa from patients with chronic *S. aureus* airway infection to visualize NETs by immuno-fluorescence and confocal microscopy. Next, we determined nuclease activity of sequential and isogenic clinical *S. aureus* CF isolates by DNase agar plates and a FRET-based assay to evaluate nuclease activity. Since the expression of nuclease confers escape from NET-mediated killing to *S. aureus*, NET-killing assays of isolates with different nuclease activity were performed. To confirm the specific effect of nuclease regarding NET-mediated killing, a clinical *S. aureus* isolate with low nuclease activity was transformed with a plasmid that expresses wild-type nuclease, and tested in the NET-killing assay. To verify the role of nuclease *in vivo*, two independent sputa of an individual CF patient were used for RNA sequence analysis.

Our data revealed, that (i) NET-structures were visible in CF sputa and that *S. aureus* was in close proximity to NETs, (ii) nuclease activity of isogenic sequential isolates of one individual patient increased significantly during persistence, (iii) isolates with high nuclease activity were protected against NET-mediated killing, (iv) protection against NET-mediated killing was caused by higher nuclease activity, and that (v) nuclease was highly expressed in sputa of an individual CF patient.

## Results

### Extracellular DNA Entraps *S. aureus* Bacteria in CF Sputum Samples

**Figure 1:**
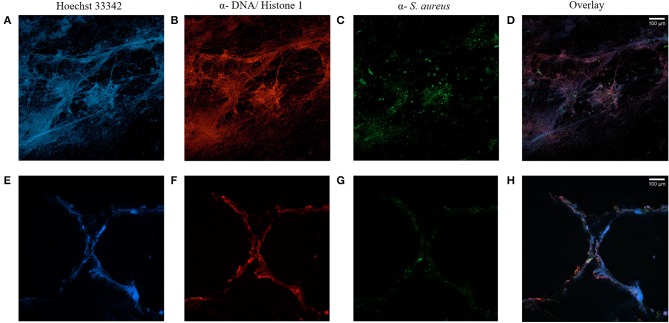
CF sputum samples visualized by immunofluorescence staining and confocal microscopy. Staining of two sputa (**A–D** and **E–H**) of two different CF patients with chronic *S. aureus* infection. Nuclei and DNA structures (blue, **A,E**) and DNA-histone-1-complexes (red, **B,F**) are visible, whereas antibody-mediated staining specifically identifies *S. aureus* (green, **C,G**). Microscopy reveals NET-structures with *S. aureus* attached to it, but also with *S. aureus* without NET-binding **(D,H)**. Subsequent changes of color balance for color intensification were performed with ImageJ.

To examine, if NET- formation occurs in the airways of CF patients with chronic *S. aureus* infection, confocal microscopy of stained sputum was performed and confirmed the presence of high amounts of NETs (Figures 1A,B,E,F). Furthermore, fluorescence microscopy revealed *S. aureus* being mostly entangled in extracellular DNA structures (Figures 1C,D,G,H), while there were also bacteria visible in an environment without NETs.

### Nuclease Activity of *S. aureus* Correlates With Long-Term Persistence

**Figure 2:**
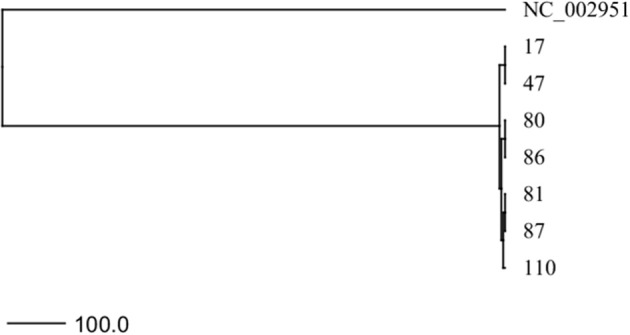
UPGMA-tree based on the cgMLST allelic profiles of 7 clinical *S. aureus* isolates. The phylogenetic tree demonstrates the relationship of 7 *S. aureus* isolates, which were collected over 14 years. The tree was drawn to scale with branches given in absolute alleles distances.

**Figure 3:**
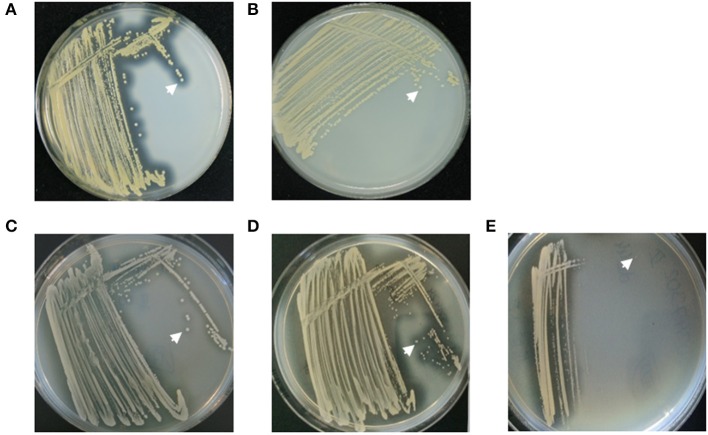
Nuclease activity in different *S. aureus* strains analyzed by DNase agar plates. **(A)** *S. aureus* strain AH1263, positive control, showed large clearing zones (white arrow) around colonies. **(B)** *S. aureus* strain AH1680 (Δ*nuc*), negative control, presented without clearing zones (white arrow). **(C)** The early CF *S. aureus* isolate showed small clearing zones around colonies (white arrow), whereas **(D)** the late isolate revealed large clearing zones around colonies (white arrow). **(E)** *S. aureus* small colony variants (SCVs) did not reveal clearing zones around single colonies (white arrow).

**Figure 4:**
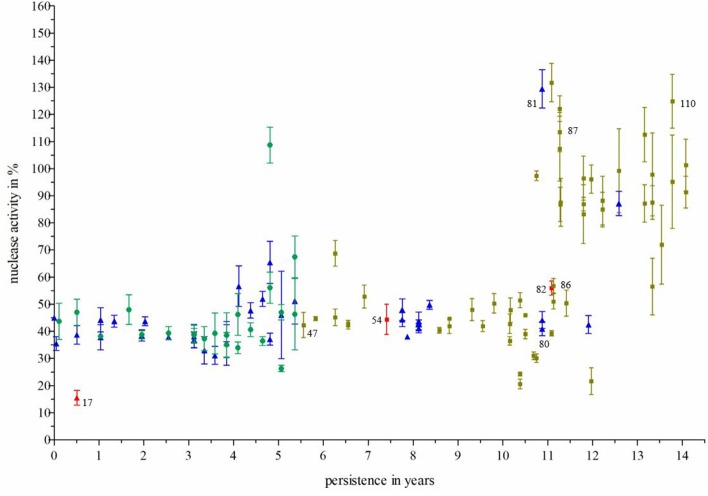
Nuclease activity of *S. aureus* isolates during persistence. Nuclease activity was measured by the nuclease FRET assay (19). *S. aureus* isolates (*n* = 111) were recovered from the nose (green circles), throat (blue rectangles) and sputum (ochre squares) from one individual CF patient during long-term persistence of 14 years. There was a significant increase of nuclease activity after 11 years. Three clinical *S. aureus* isolates (17, 54, and 82; marked red) were selected for *nuc* expression analysis via qRT-PCR. Two isolates were further analyzed in NET-killing assays and used for transformation experiments representing a strain pair with low nuclease activity (no. 17) and high nuclease activity (no. 81), respectively. Technical replicates in the FRET assay: *n* = 3, biological replicates *n* = 3. For the calculation of significance, (i) isolates were grouped into nose, throat, and sputum samples and compared by the one- way analysis of variance (ANOVA) and Bonferroni's Post-Test, resulting in: nose vs. sputum **p ≤ 0.01; nose vs. throat had no significance, throat vs. sputum ***p ≤ 0.001; (ii) regardless of sample site, isolates were grouped into early (0–7.039 years of persistence) and late (7.039–14.0.78 years of persistence). Both groups were compared using a two-tailed, unpaired student's *t*-test (result: ***p ≤ 0.001).

**Figure 5:**
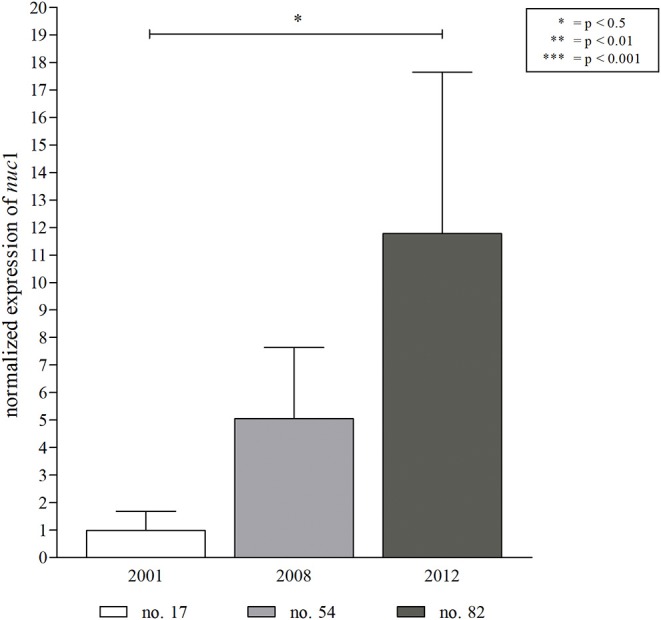
Expression of *S. aureus nuc*1 in an early, intermediate and late *S. aureus* isolate from patient 1. For the assessment of *nuc* expression, isolates (Figure 4, in red) were grown in BHI medium until mid-logarithmic growth phase before RNA extraction. Expression of *nuc*1 increased significantly over time of *S. aureus* persistence. The obtained results are in line with the nuclease FRET assay detecting low nuclease activity for isolate no. 17 (2001, 15.9%), an intermediate activity for no. 54 (2008, 45.5%), and a high nuclease activity for the late isolate no. 82 (2012, 56.84%). Statistical analysis: two-tailed, unpaired student's *t*-test, error bars represent SD. Technical replicates *n* = 2, biological replicates *n* = 3.

To assess adaptation of *S. aureus* during long-term persistence regarding nuclease activity, 111 *S. aureus* isolates, which were recovered during 14 years of persistence from the airways of an individual CF patient (CF patient 1, Table 1), were further investigated. These isolates belonged predominantly to *spa*-type t617, showing that this patient was infected with one dominant *S. aureus* clone throughout persistence. Clonality of isolates was also confirmed by whole genome sequencing of 7 selected isolates (Figure 2). An initial approach for the categorization of nuclease activity of these *S. aureus* isolates was conducted on DNase agar plates (Figures 3A–E) (18) revealing an increase of nuclease activity in late compared to early *S. aureus* isolates (Figures 3C,D, Figure Supplementary 1). Moreover, the capability to degrade DNA was also seen to be phenotype-related in *S. aureus*. Strains with a normal phenotype showed larger clearing zones around colonies compared to small colony variants (SCVs), where no DNA degradation was detected around single colonies (Figure 3D). To quantify nuclease activity, all 111 *S. aureus* isolates were subjected to the nuclease FRET assay (19) showing a significant increase of nuclease activity in late isolates after 11 years of persistence (Figure 4). These results were confirmed by qRT-PCR analysis, which demonstrated a significant increase of *nuc*1 expression after 11 years of *S. aureus* persistence (Figure 5). Also, *nuc*2 expression revealed a significant increase after 7 years of persistence (Figure Supplementary 2). However, *nuc*2 expression (Figure Supplementary 2) was much lower than *nuc*1 expression (Figure 5).

**Table 1:** Clinical *S. aureus* isolates of CF patients used in this study.

**Patient**	**Year of recovery**	**Sample site**			***spa*-type**
		**Nose**	**Throat**	**Sputum**	
1	2001	1,3	2,4,5,17		t617
1	2002		6		t499
1	2002	7	8,9,16		t617
1	2003		11		t499
1	2003	10,12,15	13,14		t617
1	2004	19,20,22,25	18,21,23,24		t617
1	2005	28–31,33	26,27,32		t617
1	2005		34		t930
1	2006	35,37,38,41,42,44,45	36,39,40,43,46	47	t617
1	2007			49	t930
1	2007			48,50–52	t617
1	2008			53	t930
1	2008			54	t034
1	2009		55–61	62	t617
1	2010			63–66	t617
1	2011			67–70,72,74,75	t617
1	2011			71	t002
1	2011			73	t230
1	2012		79–81	76–78,82–92	t617
1	2013		101	93–95, 97,99,100	t617
1	2013		96		t930
1	2013			98	t121
1	2014			102	t1459
1	2014			103–108	t617
1	2015			109–111	t617
2	2016			881*,912*	t034

Isolates are numbered in chronological order of recovery. The year of recovery is presented, as well as the sample site of which isolates were recovered from the patient and the identified spa-type. NET killing experiments were performed with isolates from two different CF patients. Isolate no. 17 and no. 81 with low and high nuclease activity were recovered from one individual CF patient (underlined). Isolate no. 881 and 912 (marked with *) with low and high nuclease activity were recovered from another CF patient, respectively (Figure Supplementary 3). Isolate no. 17 was further transformed with the pCM28nuc plasmid and underwent NET killing analysis, whereby no. 81 served as the comparison strain with high nuclease activity.

### Inverse Expression Pattern of Nuclease and *agr*

**Figure 6:**
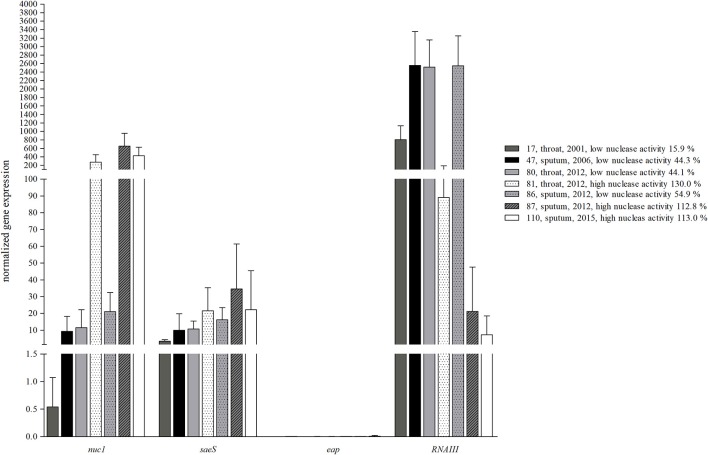
Inverse expression pattern of nuclease and *agr*. Sequential clinical *S. aureus* isolates from one individual CF patient were grown in BHI until mid-logarithmic growth phase to determine *nuc1*, the potential *nuc* regulator *saeRS* (represented by *saeS*), *eap* as another *saeRS* regulated virulence factor and *RNAIII* as the effector gene of the *agr* regulon. *Nuc* expression was independent of *saeS*, but was inversely correlated with *agr*, while *eap* was only very low expressed. Isolates with low nuclease activity (17, 47, and 86) revealed high *RNAIII* expression, whereas isolates with high nuclease activity (81, 87, and 110) low *RNAIII* expression, respectively. Technical replicates *n* = 3, biological replicates *n* = 4.

Seven clinical *S. aureus* strains (Figure 4: 17, 47, 80, 81, 86, 87, and 110) from one individual CF patient were subjected to qRT-PCR to analyze expression of *nuc*1 in relation to potential regulators of *S. aureus* nuclease (Figure 6). The results of *nuc*1 expression were in accordance to the results of nuclease activity by the nuclease FRET assay. Since *S. aureus* nuclease was shown to be SaeRS-regulated (20), transcription of *saeS* was determined for clinical isolates with low and high nuclease activity. Surprisingly, no alterations in *saeS* expression were observed for *S. aureus* isolates dependent of either low or high nuclease expression (Figure 6). Also, *eap*, a protein, which has been shown to be under the control of *saeRS* (21), was only very low expressed without any association to nuclease expression in all 7 clinical isolates. Interestingly, RNAIII, the effector molecule of the major virulence regulator *agr* (22), was higher expressed in *S. aureus* isolates with low *nuc*1 expression, whereas decreased RNAIII expression was observed in isolates with high nuclease expression (Figure 6) indicating an inhibiting role of *agr* in nuclease expression in the clinical isolates. Interestingly, whole genome sequencing revealed two subsequent non-synonymous SNPs in *agrA* of those *S. aureus* isolates with decreased RNAIII transcription and increased nuclease activity (Table 2).

**Table 2:** Non-synonymous SNPs in agrA.

**Gene**	**Target**	**Rel. position**	**NC_002951**	**Variant AA**	**17, 47**	**Isolates with variants**	
						**80, 86**	**81, 87, 110**
*agrA*	SACOL2026	409	G	D → Y	G	**T**	**T**
*agrA*	SACOL2026	520	C	H → Y	C	C	**T**

*Two subsequent non-synonymous SNPs were discovered in agrA of the clinical S. aureus isolates. Nuclease and RNAIII activity inversely correlated with the presence of both mutations*.

### Nuclease Activity Differs in CF Patients

**Figure 7:**
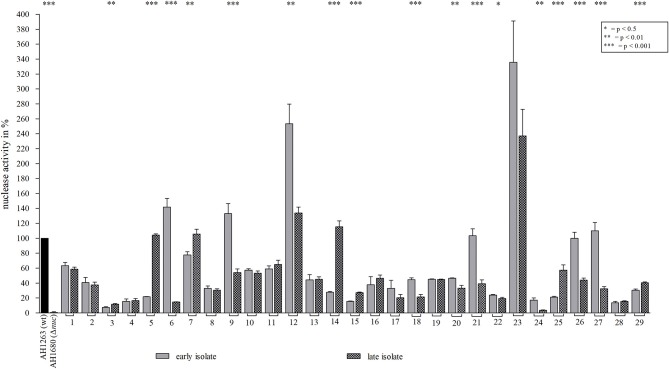
Nuclease activity of different early/late *S. aureus* strain pairs. Nuclease activity of 29 *S. aureus* strain pairs (early, late) from different CF patients (23) were analyzed by the nuclease FRET assay. Regarding nuclease activity in late isolates, an increase was observed in 7 late strains (strain pairs 3, 5, 7, 14, 15, 25, and 29), a decrease in 10 (strain pairs 6, 9, 12, 18, 20, 21, 22, 24, 26, and 27) and an unchanged activity in 12 late strains. *S. aureus* strains AH1263 and AH1680 (Δ*nuc*) served as positive and negative control, respectively. Statistical comparisons: two-tailed, unpaired student's *t*-test, error bars represent SD. Technical replicates *n* = 3, biological replicates *n* = 3.

To assess, if an increase in nuclease activity is a common adaptation pattern in other CF patients with long-term *S. aureus* persistence, 29 *S. aureus* strain pairs (early and late isolate) of different CF patients were analyzed by the nuclease FRET assay. Seven of these 29 late *S. aureus* isolates revealed a significant increase of nuclease activity (Figure 7), whereas in 10 late isolates a significant decrease of nuclease activity and in 11 late strains, no change in nuclease activity was observed (Figure 7).

### Higher Nuclease Activity Facilitates Increased Survival of *S. aureus* Interacting With NETs

**Figure 8:**
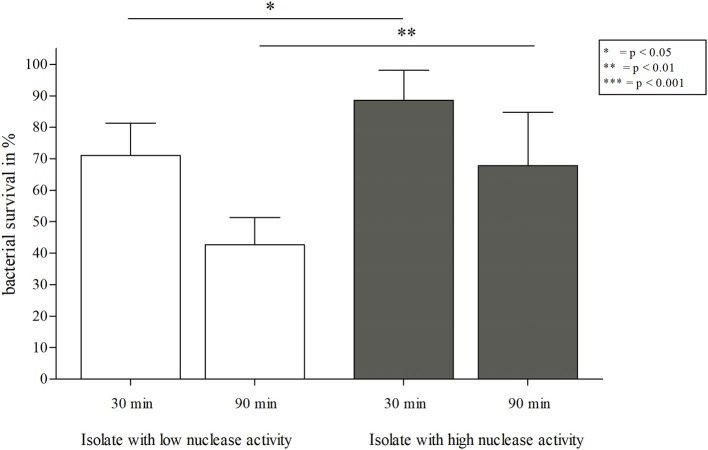
NET-killing assay with *S. aureus* isolates from patient 1 with low/high nuclease activities. Survival rates (in %) after 30 and 90 min of incubation with NETs are depicted. A significant decrease of bacterial survival was observed for the (early) isolate with low nuclease activity (Figure 4, isolate 17) after 30 and 90 min of incubation with NETs in comparison to the corresponding (late) isolate with high nuclease activity (Figure 4, isolate 81). Statistical comparison of both *S. aureus* strains depending on incubation time: two-tailed, unpaired student's *t*-test, error bars represent SD. Statistical comparison of single *S. aureus* strains: two-tailed, paired student's *t*-test. Technical replicates *n* = 2, biological replicates *n* = 6.

**Figure 9:**
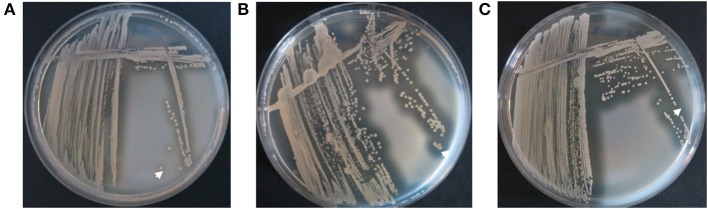
Analysis of nuclease activity on DNase agar plates after the transformation of the early *S. aureus* isolate with low nuclease activity of patient 1 with the plasmid pCM28*nuc*. **(A)** The early *S. aureus* isolate with low nuclease activity (Figure 4, isolate17) shows minimal clearance zones (white arrow) around colonies. **(B)** The pCM28*nuc*-transformed early *S. aureus* isolate exhibits large clearance zones, **(C)** similar to the corresponding late *S. aureus* isolate with high nuclease activity (Figure 4, isolate 81).

**Figure 10:**
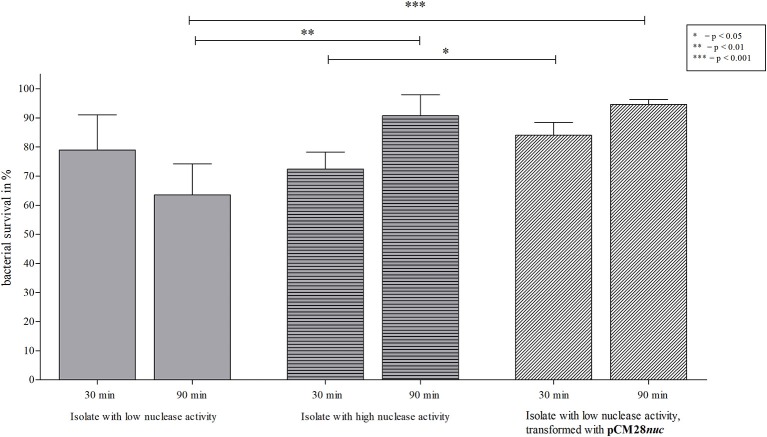
NET-killing assay with an early *S. aureus* isolate with low nuclease activity of after transformation with pCM28*nuc*. Survival rates (in %) after 30 and 90 min of incubation with NETs are shown. A significant decrease of bacterial survival after 90 min is apparent for the isolate with low nuclease activity (Figure 4, isolate 17), compared to both isolates with enhanced nuclease activity. After 30 min, the survival rate for the isolate with high nuclease activity (Figure 4, isolate 81) is significantly lower than for the transformed strain. After 90 min, no difference in survival was observed for both *S. aureus* strains with high nuclease activity, but enhanced survival was shown for the two strains with high nuclease activity compared to the strain with low nuclease activity. Statistical comparison of *S. aureus* strains depending on incubation time: two-tailed, unpaired student's *t*-test, error bars represent SD. The comparison of single *S. aureus* strain pairs revealed no statistical differences (two-tailed, paired student's test). Technical replicates *n* = 2 and biological replicates *n* = 7 were performed.

Since secretion of nuclease facilitates evasion from NETs (17), we assessed survival of *S. aureus* isolates with high and low nuclease activity in interaction with NETs. After 90 min, the *S. aureus* isolate with low nuclease activity (Table 1, no. 17 and 881) was significantly more affected by NET-mediated killing than the isolate with high nuclease activity (Table 1, no. 81 and 912; Figure 8, Figure Supplementary 3). In detail, the survival of the isolate with low nuclease activity was 54.7% in comparison to 74.9% of survival of the isolate with high nuclease activity (Figure 8). To confirm the role of nuclease in the context of NET-mediated killing on bacterial survival, the *S. aureus* isolate with low nuclease activity (Table 1, no. 17) was transformed with the nuclease expressing plasmid pCM28*nuc* (Table 3), which resulted in increased nuclease activity for the transformed strain (Figure 9). Similar to our previous results (Figure 8, Figure Supplementary 3), the CF isolate with low nuclease activity was killed to a significant higher extent than the corresponding late isolate with high nuclease activity after 90 min of NET interaction (Figure 10). In line with these results, the NET-killing capacity of the transformed strain was comparable to the late isolate with high nuclease activity (Figure 10). Interestingly, after 90 min of co-incubation with NETs, the bacterial survival of the transformed isolate with former low nuclease activity was higher than after 30 min of co- incubation, indicating that the high activity of nuclease conferred protection against NET-mediated killing and even allowed replication of bacteria as also observed for the late isolate in Figure 10.

**Table 3:** *S. aureus* reference strains, plasmid and plasmid transformed strain used in this study.

**Strain or plasmid**	**Description**	**References**
AH1263	USA300 CA-MRSA ErmS (LAC), wild type strain	(24)
AH1680	AH1263 *nuc*:LtrB, Δ*nuc* mutant of wild type AH1263	(25)
pCM28*nuc*	*nuc*-complementing vector with chloramphenicol resistance	(25)
17 [pCM28*nuc*]	*S. aureus* strain with low nuclease activity transformed with pCM28*nuc*	This work

### Nuclease Expression *in vivo* in Sputa

**Figure 11:**
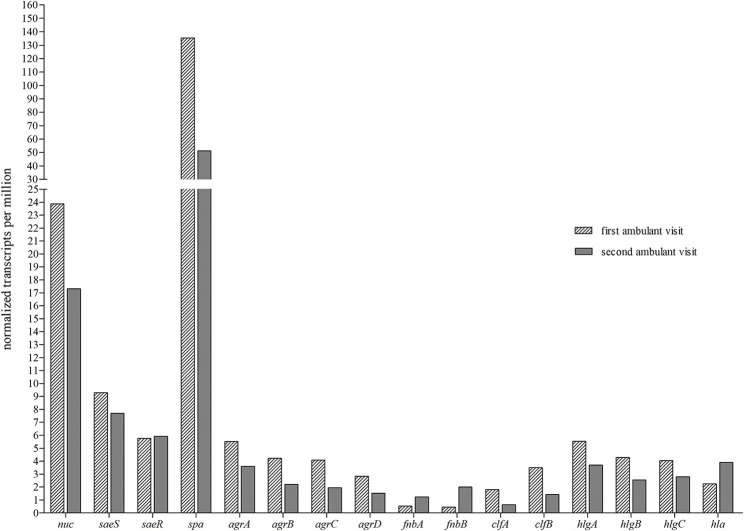
RNA-Seq analysis of *in vivo* expression of nuclease and other important *S. aureus* virulence regulators and genes in sputa from one individual CF patient. Sputum samples were collected from a chronically infected CF patient at two visits and subjected to RNA-Seq analysis. Expression levels of nuclease (*nuc*), virulence regulators (*saeS, saeR, agrA, agrB, agrC*, and *agrD*), protein A (*spa*), fibronectin-binding proteins (*fnbA*, f*nbB*), clumping factor proteins (*clfA, clfB*), the subunits for γ-hemolysin (*hlgA, hlgB*, and *hlgC*) and α-hemolysin (*hla*) are shown as normalized transcripts per million (TPM). TPM levels of presented genes were normalized against TPM values 600 of the *S. aureus* housekeeping genes *aroE* and *gyrB*.

To determine the role of nuclease expression *in vivo*, we collected two different sputa from one individual CF patient, who was persistently infected by *S. aureus*, and subjected this sputum to RNA-Seq analysis (Figure 11). The comparison of normalized transcription of *nuc* and other important virulence regulators and virulence factors against housekeeping (hk) genes revealed a high relative expression of *nuc* in both sputa, which was only surpassed by the expression of protein A (*spa*). The third highest transcript levels were obtained for *saeS*, which has been shown to be a regulator of *nuc* (20), while levels of adhesins (*fnbA, fnB, clfA*, and partially *clfB*) were low in both analyzed sputa (Figure 11). The obtained results of *in vivo* RNA-Seq analysis indicate the importance of nuclease during *in vivo S. aureus* airway infection.

## Discussion

Airway infections in CF lung disease are associated with strong inflammatory responses characterized by a domination of neutrophils (6), which can use different strategies to combat invading pathogens. Recently, NET-formation, also known as NETosis, within the airways of CF patients gained interest and it has been shown that abundant NETosis and NET- related markers in CF airway fluids occur (14, 26, 27). Most of these studies evaluated NET-formation in regard to *P. aeruginosa* airway infection in CF patients (15, 27–29). To our knowledge, there are no data about NET-formation and *S. aureus* in the context of CF. Therefore, we assessed, if sputum of patients with chronic *S. aureus* airway infections contain NETs. Several sputa of CF patients were stained for extracellular DNA structures characteristic for NETs and for *S. aureus*. As shown in two sputum samples exemplarily (Figure 1), *S. aureus* bacteria were visible in close proximity to NET- structures, but also without relation to NETs indicating that not all *S. aureus* bacteria are entangled and killed by NETs in CF sputa and that some isolates may have escaped NET-mediated killing. Since secreted *S. aureus* nuclease facilitates escape from NETs (17), we hypothesized that nuclease activity in long-persisting isolates would differ in comparison to early isolates. Therefore, we tested nuclease activity of more than 100 sequential *S. aureus* isolates, which were collected during persistence in the airways of one individual CF patients during a 14-year period. Our results showed, that isolates with increased nuclease activity occurred not earlier than after 11 years of persistence and were selected during the later years of persistence (Figure 4). These results were confirmed by qRT-PCR of selected strains with differential nuclease activity (Figure 5). Isolates with increased nuclease activity were mostly isolated from sputa, while isolates with low nuclease activity were more likely isolated from nasal and throat swabs (Figure 4). The fact, that isolates with high nuclease activity were more likely isolated from sputa indicates that there is a greater chance of selection of isolates with high nuclease activity at a site where more neutrophils are present, which most likely perform NETs. However, after 11 years most *S. aureus* isolates displayed higher nuclease activity indicating a survival advantage and pathoadaptation of these isolates to the NET-rich airways in CF as it has been suggested also by Rahman and Gadjeva (30).

In addition, isolates with SCV phenotype revealed less nuclease activity compared to normal growing isolates. Such data are in line with our earlier results showing that SCVs are characterized by a down- regulated metabolism, which facilitate long-term persistence (31, 32). Therefore, increased nuclease activity does not seem to be of importance for long-term survival for SCVs. Moreover, since SCVs mostly occur in mixed cultures with normal *S. aureus*, the secreted nuclease of the normal *S. aureus* might protect also SCVs from NET-mediated killing. Such assumption should be assessed in further NET-killing experiments with mixed cultures of normal and *S. aureus* SCVs.

As we investigated early/late *S. aureus* isolates from 29 different CF patients, only in the late isolates of 7 CF patients nuclease activity was significantly increased. However, considering the fact that nuclease increase in the sequential isolates of the described patient occurred only after 11 years of persistence, many of the studied late isolates might not have persisted long enough in the airways in order to adapt to NET-formation. Moreover, an increase in nuclease activity especially occurred in *S. aureus* isolates recovered from sputa. Since not all late isolates of the tested 29 strains pairs were collected from sputa (Table Supplementary 1), this also might have influenced the results regarding the neutrophil domination in deeper CF airways, that is accompanied by a massive release of inflammatory agents (6, 33–35). Based on our findings, the observed enhancement of nuclease might be a result of a higher selective pressure for *S. aureus* present in lower airways compared to those that are present in nose or throat. Recently, Berends et al. showed resistance of *S. aureus* against NET-mediated entrapment and killing by the secretion of *S. aureus* nuclease (17). Therefore, we tested survival of NET-mediated killing of *S. aureus* isolates with high in comparison to low nuclease activity showing that *S. aureus* isolates with high nuclease activity were less killed by NETs compared to isolates with low nuclease activity. Such results indicate that *S. aureus* isolates with high nuclease activity were most likely selected in the airways of CF patients with high abundance of NET formation, which facilitate survival of *S. aureus* in this hostile environment. The beneficial role of high nuclease expression was confirmed by transformation of a clinical CF isolate with low nuclease with the nuclease expressing vector pCM28*nuc*. Survival of the transformed *S. aureus* strain during NET-killing was significantly increased compared to the corresponding isolate with low nuclease activity.

The fact that nuclease expression was also high in two *in vivo* sputum samples from one individual CF patient with persistent *S. aureus* airway infection as assessed by RNA-Seq analysis underscores the role of nuclease for the *in vivo* situation. Elucidating the transcriptome of *S. aureus* in the nasal niche by RNA-Seq analysis (36), the comparison of transcript levels of *S. aureus* recovered from CF sputum demonstrated higher expression of nuclease and the nuclease-regulating *saeS* component (20) compared to the nasal *S. aureus* isolate. The increase of *saeS* and *nuc* in CF compared to nasal isolates (36) underscores our hypothesis of *S. aureus* combating neutrophil-mediated eradication by the up-regulation of nuclease during adaptation to the highly inflammatory CF airways.

If this high nuclease activity has an impact on macrophage viability during NET formation, especially regarding the concerted action of nuclease and adenosine synthase A as shown recently by Thammavonsga et al. (37), is an interesting aspect, which should be investigated in future experiments.

A first insight about the molecular mechanism of the increase in nuclease activity in the clinical isolates is given by the detected SNPs within *agrA*, which most likely caused down-regulation of RNAIII expression in late clinical isolates. *AgrA*-specific mutations have been previously shown by others (38, 39) to be responsible for a change in *agr* activity. Surprisingly, *saeRS*, which has been shown by others to positively regulate *nuc* (20, 21, 40) had no apparent impact on nuclease activity in these clinical isolates.

In conclusion, our data underline the importance of *S. aureus* nuclease activity during long-term airway infection of CF patients. In sputa of patients with chronic *S. aureus* infection, NET formation with entrapped *S. aureus* was apparent. In many CF patients, pathoadaptation of long-term persisting *S. aureus* isolates to the airways, where abundant NET formation is observed, was accomplished via increased nuclease activity. High nuclease activity conferred a survival advantage to *S. aureus* during NET-killing and high nuclease expression was determined in *in vivo* sputa from a CF patient chronically infected by *S. aureus*.

## Materials and Methods

### Patients' and Volunteers' Specimens

The usage of sputum of CF patients and blood samples was approved by the Ethical Committee of the University Hospital Münster (no. 2018-466-f-S). Informed consent was given for blood samples by healthy volunteers and for sputa by CF patients.

### Bacterial Strains and Growth Conditions

Clinical *S. aureus* isolates with the same or closely related *spa*-types (*n* = 111) from one individual CF patient, cultured from throat, nose, or sputum samples collected during a period of 14 years were used (Table 1). To assess adaptation regarding nuclease activity during persistence in more CF patients, *S. aureus* strain pairs of CF patients consisting of early and late isolates with at least 5 years of *S. aureus* persistence with identical or closely related *spa*-types were analyzed (Table Supplementary 1). These strain pairs were previously characterized in terms of virulence factor adaptation (23). All CF isolates and reference strains (Table 3) were cultivated on Columbia 5% blood agar plates (BD). Overnight cultures were grown in Brain Heart Infusion medium (BHI, Difco) at 37°C and 160 rpm. B2 medium was used for *S. aureus* plasmid transformation [modified after (41)]. To assess nuclease activity in qRT-PCR and NET-killing experiments, overnight cultures of *S. aureus* isolates in BHI were cultivated in fresh BHI medium for 4 h until mid-logarithmic growth phase starting from an OD_578nm_ of 0.1. For the nuclease FRET assay, *S. aureus* was cultured in 96-well-plates under the same conditions. The pCM28*nuc* plasmid was maintained in *S. aureus* AH1773 on tryptic soy agar (TSA, Difco) supplemented with 10 μg/ml chloramphenicol (CN).

### *spa*-Typing of Clinical CF *S. aureus* Isolates

Molecular typing of sequential *S. aureus* isolates (Table 1) recovered from airway specimens (nose, throat, sputum) of an individual CF patient were performed by *spa-*typing (32, 42).

### Whole Genome Sequencing of Clinical CF *S. aureus* Isolates

The clonal relationship of 7 *S. aureus* strains recovered from one individual CF patient (Table 1) was determined by whole genome sequencing (WGS) and subsequent core genome multilocus sequence typing (cgMLST). Genomic DNA of *S. aureus* isolates was purified using the MagAttract HMW DNA kit (Qiagen) following the manufacturer's instructions with the addition of 120 U Lysostaphin (Sigma-Aldrich). Clinical isolates were sequenced using Illumina technology and Nextera XT version 2 chemistry, with a 250-bp paired-end protocol on a MiSeq sequencer (Illumina). Quality trimming of fastq files (average base quality of 30, aiming for 100-fold coverage) and *de novo* assembly using SKESA (PMID: 30286803) were performed with SeqSphere^+^ (version 6; Ridom GmbH, Münster, Germany) as described recently (43). Only genomes harboring ≥95% cgMLST targets of the *S. aureus* cgMLST scheme (44) passed quality control; otherwise, sequencing was repeated. For tree building using the unweighted pair group (UPGMA) method, the allelic profiles of up to 1, 861 core genome multilocus sequence typing (cgMLST) targets were used as described previously (45). To investigate molecular mechanisms associated with nuclease activity, the SeqSphere^+^ software was used for SNP calling within the *agrA* gene.

### Nuclease Test on DNase Agar

CF isolates and laboratory strains were streaked on DNase agar plates (Oxoid) and incubated for 17 h at 37°C. DNase agar plates were flooded with 1 N hydrochloric acid solution and evaluated qualitatively by the size of clearing zones around bacterial colonies as a result of DNA degradation by *S. aureus* nuclease. *S. aureus* strains AH1263 (wild-type) and AH1680 (Δ*nuc*-mutant) were used as negative and positive control, respectively (Table 3).

### Nuclease FRET Assay

For the preparation of overnight cultures, single colonies of CF isolates and laboratory strains were inoculated in BHI medium in a 96-well-flat-bottomed microtiter plate with lid. AH1263 and AH1680 (Table 3) were used as negative and positive controls. The plate was incubated overnight for 16–18 h at 37°C using the TECAN Multireader. Overnight cultures were diluted to an OD_578nm_ of 0.1 in fresh BHI medium in a new 96-well-plate and incubated for another 4 h at 37°C in the TECAN Multireader. Analysis of nuclease activity in *S. aureus* strains was accomplished by the use of a molecular beacon as described recently (19). In brief, for analysis of nuclease activity, bacterial supernatants were collected and applied in 1:200 dilutions together with the molecular beacon (final concentration: 0.1 μM) into a black 96-well-plate. Nuclease activity was determined as a kinetic measurement in 30 min intervals with excitation at 485/20 nm, emission at 528/20 nm under fast orbital shaking. Results were evaluated with the Gen5 best fit method. Nuclease activity was calculated in a growth-dependent manner to exclude impacts on actual nuclease activity by differing growth capacities of *S. aureus* CF strains in BHI medium. Growth- fitted nuclease activity was achieved by relative nuclease activity multiplied against a factor resulting from OD_578nm_ values of the analyzed CF strain, obtained after 4 h of incubation in fresh BHI, against the OD_578nm_ value of AH1263 (100% control).

### qRT-PCR

Expression of both nucleases (*nuc*1, *nuc*2), *saeS, eap*, and *RNAIII* was assessed in the mid-logarithmic growth phase in BHI. RNA isolation, cDNA synthesis, and qRT-PCR were conducted as described (46). RNA samples were evaluated for successful DNA elimination by PCR using *S. aureus gmk* (guanylate monophosphate kinase) primers. The house-keeping genes *aroE* (Shikimate dehydrogenase NADP/H) and *gyrB* (DNA gyrase subunit B) served for normalization of nuclease gene expression. All primers used are described in Table 4.

**Table 4:** Primers used in qRT-PCR experiments.

**Target**	**Sequence**	**References**
*aroE*-fwd	5′-CTATCCACTTGCCATCTTTTAT-3′	(46)
*aroE*-rev	5′-ATGGCTTTAATATCACAATTCC-3′	
*gmk*-fwd	5′-AAGGTGCAAAGCAAGTTAGAA-3′	This work
*gmk-*rev	5′-CTTTACGCGCTTCGTTAATAC-3′	
*gyrB*-fwd	5′-AATTGAAGCAGGCTATGTGT-3′	(46)
*gyrB*-rev	5′-ATAGACCATTTTGGTGTTGG-3′	
*nuc1*-fwd	5′-AAGCTTTAGTTCGTCAAGGC-3′	This work
*nuc1*-rev	5′-TGAATCAGCGTTGTCTTCGC-3′	
*nuc2*-fwd	5′-TGGATGGTGATACATTTATTGC-3′	This work
*nuc2*-rev	5′-GTTTCACCGTTTCTGGCG-3′	
*RNAIII-*fwd	5′-ttcactgtgtcgataatcca-3′	(47)
*RNAIII*-rev	5′-tgatttcaatggcacaagat-3′	
*eap*-fwd	5′-AAGCGTCTGCCGCAGCTA-3′	(48)
*eap*-rev	5′-TGCATATGGAACATGGACTTTAGAA-3′	
*saeS*- fwd	5′-tcgaacgccacttgagc-3′	This work
*saeS*-rev	5′-ctatcgacattgctattagc-3′	

### Transformation of a CF *S. aureus* Isolate With Low Nuclease Activity

One selected *S. aureus* strain (no. 17, year 2001, throat, Table 1) with low nuclease activity was transformed with the pCM28*nuc* plasmid, containing the major *S. aureus* nuclease (*nuc*1) sequence. For the preparation of electro-competent cells, the CF isolate was cultivated in B2 medium (41) overnight. The culture was then diluted to OD_578nm_ of 0.5 in fresh B2 medium and incubated at 37°C, 160 rpm to OD_578nm_ of 0.6. Bacterial growth was stopped on ice and a pellet was harvested by centrifugation (3,000 × g for 10 min at RT). The pellet was consecutively washed in decreasing volumes of 4°C cold water before 4°C cold 10% glycerol was applied. For transformation, electro-competent cells of the CF isolate were mixed with 5 μl of the purified pCM28*nuc* plasmid (NEB Monarch Plasmid Miniprep Kit). Electroporation was executed by the Ec2 program of the BIORad MicroPulser Electroporator (Pulse 2.5 kV, number of impulse 1) in a 0.2 cm electroporation cuvette. After incubation in B2 medium for 2 h at 37°C and 350 rpm, screening for plasmid-positive colonies was performed by plating the mixture on TSA agar with 10 μg/ml chloramphenicol. The TSA plates were incubated at 37°C for 42 h. Colonies were verified for the acquisition of the pCM28*nuc* plasmid by PCR with nuclease specific primer (Table 4) after plasmid purification (NEB Monarch Plasmid 344 Miniprep Kit). Nuclease activities of the original and transformed CF isolate were compared by DNase agar plates as well as by the nuclease FRET assay (data not included).

### NET-Killing Assay

Isogenic *S. aureus* isolates with low and high nuclease activity were analyzed using the NET-killing assay (17). Human neutrophils from healthy individuals were isolated from heparin-anticoagulated blood by gradient separation using PolymorphPrep (Abbott Diagnostics Technologies AS). Remaining erythrocytes were lysed with sterile water after the neutrophil pellet was purified with magnesium- and calcium-free 1 x Dulbecco's Phosphate Buffered Saline (DPBS). Isolated neutrophils were suspended in RPMI medium (Sigma-Aldrich) with 2% heat-inactivated fetal bovine serum (FBS, Fiebig). In 24-well-plates, 2 × 10^6^ neutrophils/ml were incubated with 10 μg/ml cytochalasin D (Sigma-Aldrich) and 25 nM phorbol 12- myristate 13-acetate (PMA, Sigma-Aldrich) for 20 min at 37°C + 5% CO_2_ prior infection. With a multiplicity of infection (MOI) of 2, *S. aureus* strains were applied as 4 x 106 bacteria/ml to RPMI medium (growth control) and activated neutrophils, respectively, and incubated at 37°C + 5% CO_2_ for 30 min and 90 min. Bacterial strains were pre-cultured in BHI (overnight, for mid-logarithmic growth phase) as described above. The survival rates for each S. *aureus* strain were calculated in % by comparison of CFUs on blood agar plates of bacteria interacting with NET-forming neutrophils vs. bacteria in medium.

### Sputum Staining

Visualization of DNA-related structures and *S. aureus* bacteria in sputa from different CF patients was realized with immuno-fluorescence staining (49, 50). After expectoration, fresh sputum was immediately streaked onto poly-D-lysine-coated slides and fixed in 4% methanol-free formaldehyde for 15 min. Fixed sputum was kept hydrated either in 50 ml reaction tubes with PBS or in a humidity chamber during the immuno-fluorescence staining process. After permeabilization with 0.5% Triton-X, the CF sputum was covered with blocking buffer (1% bovine serum albumin, 10% goat serum, 0.3 mol glycine, 0.1% Tween 20 in PBS) for 20 min to avoid non-specific binding of antibodies. Subsequently, the sputum was incubated with rabbit α-*S. aureus* (MyBioSource; 1:2,000 diluted) in blocking buffer for 45 min at room temperature (RT). After washing in fresh PBS, the corresponding secondary antibody α-rabbit Alexa Fluor 488 (Invitrogen, A11070; 1:500 diluted in blocking buffer) was added and incubated for 45 min at RT in the dark. The slide was washed and covered with blocking buffer for another 10 min. A second primary antibody, mouse α-DNA/Histone1 antibody (Merck; 1:1,100 diluted), was applied for 45 min at RT in the dark to visualize NET-specific DNA- structures. After washing in PBS, the secondary antibody α-mouse Alexa Fluor 568 (Invitrogen; 1:376,500 diluted) was added together with Hoechst 33342 (Invitrogen; 1:20,000 diluted) and incubated for 45 min at RT in the dark. Prolong Antifade Mountant (Invitrogen) was applied on the slide, covered by a 24 × 60 mm glass cover slip, and incubated overnight at RT. The slides were examined for NET-structures and *S. aureus* bacteria by fluorescence microscopy, using the ZEISS Observer Z1 microscope with a Plan-Neofluar 100x/1.3 Oil RMS objective and AxioVision Rel 4.8 software. For a more detailed acquisition of images, CF sputum was further analyzed by the Zeiss LSM800 microscope equipped with a Plan-Apochromat 63x/1.4 Oil DIC M27 objective and ZEN 2.3 software.

### RNA Extraction and Sequencing Analysis of CF Sputa

Sputum of one CF patient, who is chronically colonized and infected by *S. aureus*, was collected at two different time points in 2016 and stored at −80°C until processing. Samples were gently mixed with an equivalent volume of Sputolysin (10%) and incubated during 30 min at 37°C on a ThermoMixer device. RNA extraction, library construction, sequencing analysis and data processing were conducted as described recently (36). Calculation of relative gene expression/ normalization in transcripts per million (TPM) was based on the following formula:

$\frac{TPM\;gene\;of\;interest}{\left(TPM\text{gyrB}+TPM\;\text{aroE}\right)/2}$. The *S. aureus* genes *gyrB* and *aroE* served as housekeeping genes.

### Statistical Analysis

Data were analyzed in GraphPad Prism 5. Nuclease activities of *S. aureus* isolates (*n* = 111) from one individual patient recovered from different sample sites were compared by (i) one-way analysis of variance (ANOVA) test, complemented by Bonferroni's Post-Test (ii) two-tailed, unpaired student's *t*-test. Nuclease activity and survival rates obtained by the NET-killing assays were compared by two-tailed, unpaired *t*-test. For the comparison of bacterial survival among *S. aureus* isolates, the two-tailed, paired *t*-test was used.

## Data Availability Statement

The datasets generated for this study by RNASeq can be found in the NCBI Gene Expression Omnibus (GEO) repository GSE139662, https://www.ncbi.nlm.nih.gov/geo/query/acc.cgi?acc=GSE139662.

## Ethics Statement

The studies involving human participants were reviewed and approved by Ethical Committee of the University Hospital Münster, Münster, Germany (2018-466-f-S). The patients/participants provided their written informed consent to participate in this study.

## Author Contributions

BK and SH designed the study. BK obtained the funding. SH performed and evaluated the majority of experiments and wrote the paper with the help of BK. FD established the FRET-based assay for the investigation of nuclease activity and analyzed *S. aureus* isolates from different CF patients. VS and AM performed whole genome sequencing and analyzed sequence data. DC-M and DP conducted and evaluated the RNA-Seq analysis of CF sputum. SN and CN gave theoretical and practical support with fluorescence microscopy and transformation with the pCM28nuc plasmid, respectively. NB and MK-B gave theoretical and practical support regarding the performance of NET killing assays and the immunofluorescence staining of CF sputa. JS performed *spa* typing of used *S. aureus* isolates. JG captured images of stained sputum samples using confocal microscopy under the scientific supervision of UR. AD, PK, and HS provided CF patient materials of which *S. aureus* isolates were recovered for this study, and sputum samples could be investigated on NETs. All authors read the paper.

### Conflict of Interest

The authors declare that the research was conducted in the absence of any commercial or financial relationships that could be construed as a potential conflict of interest.
