# Supplementary material for: High Nuclease Activity of Long Persisting Staphylococcus aureus Isolates Within the Airways of Cystic Fibrosis Patients Protects Against NET-Mediated Killing
Source: Front Immunol. 2019 Nov 5;10:2552. doi: 10.3389/fimmu.2019.02552 (PMC6849659; doi:10.3389/fimmu.2019.02552)
Supplement: Supplementary file 1 [file Data_Sheet_1.docx]

Supplementary Material

# Supplementary Figures and Tables

## Supplementary Tables

**Table Supplementary 1. *S. aureus* strain pairs used in this study.**

| Strain pair | Year of recovery- early isolate | Year of recovery- late isolate | Sample site (early/ late) | *spa*-type (early/ late) | Persistency in years |
| --- | --- | --- | --- | --- | --- |
| 1 | 2001 | 2008 | throat | t091 | 6,41 |
| 2 | 2000 | 2008 | sputum | t211 | 8,45 |
| 3 | 1994 | 2008 | sputum | t056/ t2565 | 13,19 |
| 4 | 2004 | 2010 | throat | t078 | 5,37 |
| 5 | 2003 | 2009 | sputum | t002 | 5,81 |
| 6 | 1995 | 2005 | sputum | t078 | 10,58 |
| 7 | 1999 | 2007 | throat/ sputum | t118 | 8,12 |
| 8 | 1995 | 2008 | throat | t012/ t021 | 13,17 |
| 9 | 2003 | 2010 | sputum | t121 | 6,58 |
| 10 | 2002 | 2010 | throat | t034 | 8,08 |
| 11 | 2001 | 2010 | sputum | t012 | 9,00 |
| 12 | 1999 | 2008 | throat | t154 | 9,32 |
| 13 | 2001 | 2007 | sputum | t034 | 5,66 |
| 14 | 1997 | 2008 | throat/ sputum | t346/ t084 | 10,91 |
| 15 | 1995 | 2008 | throat/ sputum | t080 | 13,56 |
| 16 | 1995 | 2007 | sputum | t056 | 11,80 |
| 17 | 2001 | 2008 | sputum | t084 | 6,97 |
| 18 | 2001 | 2007 | throat | t084 | 5,48 |
| 19 | 2005 | 2010 | sputum | t012 | 5,10 |
| 20 | 2002 | 2007 | sputum | t486/ t804 | 5,58 |
| 21 | 1997 | 2008 | throat | t084 | 10,31 |
| 22 | 2001 | 2010 | sputum | t127 | 8,52 |
| 23 | 2002 | 2010 | sputum | t623 | 7,52 |
| 24 | 2001 | 2007 | sputum | t922 | 5,90 |
| 25 | 1999 | 2009 | throat/ sputum | t084 | 10,03 |
| 26 | 1994 | 2003 | sputum | t002 | 8,25 |
| 27 | 2001 | 2010 | sputum | t489/ t9895 | 8,32 |
| 28 | 2003 | 2010 | throat | t619 | 6,42 |
| 29 | 2001 | 2010 | throat/ sputum | t617 | 8,44 |

These strain pairs were previously characterized (Hirschhausen & Block et al, 2013).

## Supplementary Figures





**Figure Supplementary 1. Nuclease activity of *S. aureus* isolates from one CF patient analyzed by DNase test agar plates.** Isolates were recovered over a time period of 14 years, from 2001 until 2015, in context of a longitudinal CF study. The majority of *S. aureus* isolates has low activity of nuclease until 2011, afterwards the number of isolates with an observed high nuclease activity on DNase test agar increased. Isolates recovered in the years 2013 and 2014 with no detectable capability of DNA degradation are SCVs of *S. aureus*. Results of the DNAse test agar test are in accordance to results achieved by the nuclease FRET assay (Figure 4).





**Figure Supplementary 2. Expression of *S. aureus* *nuc2* in an early, intermediate and late *S. aureus* isolate from an individual CF patient.** Growth conditions were equal to the assessment of *nuc1* (Figure 5). Similar to *nuc1*, *nuc2* expression increases during persistence of *S. aureus*. Statistical comparisons: two-tailed, unpaired student’s t-test, error bars represent standard deviation. Technical replicates n = 3, biological replicates n = 3.





**Figure S3. NET killing assay with *S. aureus* isolates from another CF patient (Table 1, marked *) with different nuclease activities.** The survival rates (in %) after 30 min and 90 min of incubation with human-derived NETs are shown. A significant decrease of bacterial survival was observed for the (early) isolate with low nuclease activity after 30 min and 90 min of co-incubation with NETs in comparison to the corresponding (late) isolate with high nuclease activity (* and **, respectively). A significant decrease within bacterial survival from 30 min to 90 min of co-incubation was seen for the isolate with low nuclease activity (***, not shown), as well as for the late isolate (*, not shown). Statistical comparison of both *S. aureus* strains depending on incubation time: two-tailed, unpaired student’s t-test, error bars represent SD. Statistical comparison of single *S. aureus* strains (not presented): two-tailed, paired student’s t-test, error bars represent standard deviation.
